# Using the Marine Rotifer *Brachionus plicatilis* as an Endpoint to Evaluate Whether ROS-Dependent Hemolytic Toxicity Is Involved in the Allelopathy Induced by *Karenia mikimotoi*

**DOI:** 10.3390/toxins10110439

**Published:** 2018-10-29

**Authors:** Yuanyuan Li, Jianfei Yu, Tianli Sun, Chunchen Liu, Yu Sun, You Wang

**Affiliations:** 1 Department of Marine Ecology, College of Marine Life Science, Ocean University of China, Qingdao 266003, China; 21160611049@stu.ouc.edu.cn (Y.L.); yujianfei@stu.ouc.edu.cn (J.Y.); 11160622014@stu.ouc.edu.cn (C.L.); 21170531126@stu.ouc.edu.cn (Y.S.); 2 National Marine Hazard Mitigation Service, Beijing 100194, China; tlsun@nmhms.org.cn; 3 Laboratory for Marine Ecology and Environmental Science, Pilot National Laboratory for Marine Science and Technology, Qingdao 266071, China

**Keywords:** allelopathic effect, hemolytic activity, ROS-dependent pathway, harmful algal blooms (HABs), *Karenia mikimotoi*, *Brachionus plicatilis*

## Abstract

The toxic effects of the typically noxious bloom-forming dinoflagellate *Karenia mikimotoi* were studied using the allelopathic experimental system under controlled laboratory conditions. The potency of intact cell suspensions with whole cells, cell-free culture filtrate in different growth phases, and lysed cells with ultrasonication were compared, and the growth and reproduction of the marine rotifer *Brachionus plicatilis* were used as endpoints to evaluate toxic differences. The intact cell suspension resulted the most significant growth inhibition, including lethality, on the growth of *B. plicatilis* (*p* < 0.05). Lysed culture medium treated with ultrasonication and the cell-free culture filtrates at either the exponential or stationary phase exhibited limited negative impacts compared to the control according to changes in the population growth rate (*r*) and survival rate (*p* > 0.05). Reproduction presented a similar tendency to change, and the number of eggs produced per individual, as well as spawning period decreased in the whole cell and lysed cell suspensions. The key parameters in the lift table include the net reproductive rate (R_0_) and the intrinsic rate of increase (*r_m_*), which were more sensitive to treatment and were significantly suppressed compared to that of the control. The addition of the ROS inhibitor N-acetylcysteine (NAC) could not change the growth or reproduction patterns. Moreover, substantial hemolytic toxicity was found in the treatment of the intact cell suspension (*p* < 0.05), while limited toxicity was found in other treatments compared to that of the control. *K. mikimotoi* was speculated to secrete allelopathic substances onto the cell surface, and direct cell contact was necessary for allelopathic toxicity in *B. plicatilis*. Reactive oxygen species (ROS)-independent hemolytic toxicity was assumed to be the explanation for what was observed.

## 1. Introduction

Harmful algal blooms (HABs), which are known for causing severe damage to the marine ecological environment and marine economy as well as to public health, are increasing in frequency, magnitude, and duration worldwide [1]. It is calculated that there are more than 100 toxic or harmful marine phytoplankton species, among which dinoflagellates are well known as the major HAB-causing species. *Karenia mikimotoi* is one of the common HAB-causing dinoflagellate species that is widely distributed in temperate and tropical shallow waters and possesses the unique feature of fish poison [2]. Toxins produced by *K. mikimotoi* have ecological implications for marine organisms, although these have not been quantified in the field [3]. It has been well documented that *K. mikimotoi* exert detrimental impacts, including lethality, on a wide range of marine life, including marine fauna [4,5] and seaweeds [6]. Three kinds of toxins were speculated to be involved in *K. mikimotoi*-induced toxicity: hemolytic toxins [7,8,9], cytotoxins [10,11] and reactive oxygen species (ROS) [12,13]. Furthermore, some species of phytoplankton were reported to produce secondary metabolites, which were called allelochemicals that play a major role in influencing the growth and development of some species more than others [14,15]. Allelopathy is one defense strategy during interference competition between different phytoplankton species [16,17]. Two different allelopathy pathways have been reported: one was toxin mediated and the other was toxin-independent, and the toxic-allelopathic pathway was more common in the natural environment compared with simply allelopathic interactions. It has been reported that *K. mikimotoi* exert noxious impacts on other species of phytoplankton [18] through the allelopathic pathway, but whether the pathway was toxin-dependent or toxin-independent or whether this allelopathic effect was species-dependent, was not well clarified.

Zooplankton represents an important link between the microbial and classic food webs in aquatic ecosystems, and the fluctuation of this species affects the sustainability of organisms at higher trophic levels [19]. The interaction between bloom-forming microalga and zooplankton was highly specific for an alga herbivore system [20] because of the complexity and variability of feeding, including the responses of ingestion, rejection, avoidance and selection in response to bloom-forming algae [21]. Moreover, reproduction impairment of zooplankton was found during the HABs. Regarding the possibly toxic mechanism, both the toxic-dependent and toxic-independent pathways were found in different alga-zooplankton interactive systems [22,23,24]. For instance, *Heterocapsa circularisquama* (Dinophyceae), which is known to have a specifically lethal effect on shellfish, was found to be toxic to a microzooplankton in a direct cell contact manner, and it released some protein-like toxic substances that were located on the cell surface and were responsible for toxicity [25]. Similar reports were found in *Alexandrium tamarense*, *Gyrodinium aureolum* [26,27], and *Favella ehrenbergii*, which were suggested to release a certain toxic substance that directly injured the co-cultured organisms. Furthermore, a reactive oxygen species (ROS) was evidenced to underlie the toxic mechanism induced by noxious dinoflagellates. In fact, ROS was a common biochemical feature of noxious or toxic bloom-forming microalga [28,29]. *Chattonella* sp. [30,31] (Oda et al., 1992, 1998) and the ichthyotoxic dinoflagellate *Cochlodinium polykrikoides* [32] generated ROS in a cell-density-dependent manner for fish killing. The generation of ROS by *K. mikimotoi* had been assumed to have close relationships with many phenomena, including histopathological changes in the gills, gastrointestinal tracts, and livers of fish [33,34]. Although several hypotheses had been proposed, the precise toxic mechanism of *K. mikimotoi* is still unclear.

Herbivorous zooplankton is an ideal first exogenous food source to fish due to its small size, slow swimming speed and ability to remain suspended in a water column. *Brachionus plicatilis* is one of the most important species of marine herbivore zooplankton and was the model organism for ecotoxicological assessment. Our previous studies had documented that the reproduction of *B. plicatilis* was sensitive to exogenous stress, and the involvement of ROS was partially responsible for reproductive toxicity [35,36,37,38]. We thus preformed the present study to further elucidate the possible toxic mechanism of *K. mikimotoi*, and the questions we were interested in were as follows: Does *K. mikimotoi* exert a hemolytic allelopathic effect on co-cultured zooplankton, and is the allelopathic effect ROS-dependent?

## 2. Results

### 2.1. Allelopathic Effects of K. mikimotoi on B. plicatilis with and without NAC Addition

**Figure 1:**
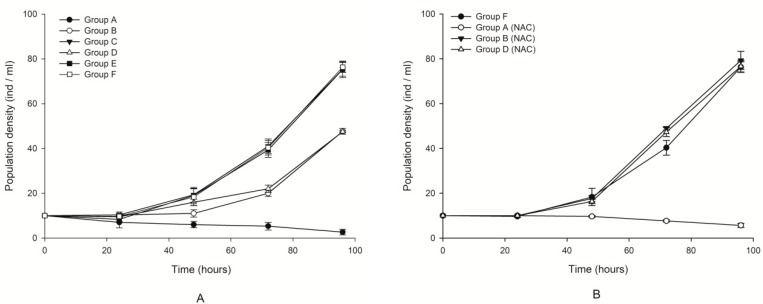
Effects of different treatment fluids on the population growth of *Brachionus plicatilis* with and without N-acetylcysteine (NAC) addition. Points were the mean ± standard error based on three replicates. (**A**) The growth curve of rotifers cultured with sterilized seawater as the control group (Group F) (□), or different treatment fluids of *Karenia mikimotoi,* Group A (●), Group B (○), Group C (▼), Group D (∆), Group E (■). (**B**) The growth curve of rotifers cultured with sterilized seawater as the control group (Group F) (●), or different treatment fluids of *K. mikimotoi* added NAC, Group A (NAC) (○), Group B (NAC) (▼), Group D (NAC) (∆).

The changes in the population density in the different treated groups are shown in Figure 1. No significant difference was observed in Groups C and E compared with the control (paired *t*-test, *p* > 0.05). This result indicated that the culture filtrates without microalgal cells at either exponential or plateau phases had little effect on the growth of *B. plicatilis*. In contrast, obvious growth inhibition (*p* < 0.05) was found in groups of lysed cell suspension that were designated as Groups B and D compared with the control. Comparatively, the most obvious growth inhibition, and even lethality, was found in Group A, which included intact microalgal cells, and the population density of the rotifers decreased steadily with the increase in exposure time age (Figure 1A). The density decreased to the lowest, even for the occurrence of mortality, approximately 100 h after exposure in this group. Therefore, the impact of microalgal fluids on the growth inhibition of rotifers was ordered by microalgal fluid with whole cells (Group A). These results suggested that whole cell suspension presented the most significant inhibition, and even lethality, on the growth of *B. plicatilis*, followed by a lysed culture medium treated with ultrasonication. The culture filtrate provoked the least impact on the rotifers. It was speculated from the result that the existence of microalgal cells were necessary for the noxious impacts of *K. mikimotoi* on rotifer, and toxins in the dissolved phase were not related to the lethal effects on the rotifers. Figure 1A also showed that the culture filtrate or lysed cell suspension at different exponential or stationary growth phase demonstrated different levels of impact on the co-culture rotifer, and those seemed exponentially more toxic than the stationary one.

NAC addition could mitigate the growth inhibition induced by microalgal fluids. We found that when NAC was added into whole cell suspension, the growth inhibition was significantly decreased compared with that without NAC addition (*p* < 0.05). However, the addition of NAC could not change the lethal effect patterns of the whole cell suspension. A similar result was found in the lysed cell suspension (Figure 1B). This result indicated that ROS only played a promoting role in the toxic effect of algae on rotifers but could not play a decisive role. It seemed that the noxiously allelopathic effect of *K. mikimotoi* was ROS-independent.

A quantitative comparison of growth inhibition was performed according to the changes in the population growth rate (*r*) (Table 1); no significant difference was found in Groups C and E compared with the control. In contrast, the population growth rates of Groups A, B and D was significantly lower compared with that of the control. However, group A showed higher toxicity than that of Groups B and D, thereby reducing the population growth rate by 0.87 compared with that of the control, Group B and Group D, which were only reduced by approximately 0.12. These results indicated that intact cell suspension has a significantly allelopathic effect on rotifers. Moreover, the population growth rate of Group C was slightly lower than that of Group E, but this difference did not reach significance (*p* > 0.05), inferring that microalga during the exponential phase were more toxic compared than microalga in the stationary phase. NAC addition obviously increased the population growth rate of Groups A, B and D compared to that without NAC addition (*p* < 0.05). However, the population growth rate of group A was still significantly lower than that of the control (*p* < 0.05). Therefore, NAC addition relieved the inhibition effects on the growth rate of *B. plicatilis* but could not play a decisive role. The results further evidenced that intact cell contact was required for the allelopathic toxicity of *B. plicatilis* and that ROS-independent allelopathy was assumed to be an explanation for the observed result.

**Figure 2:**
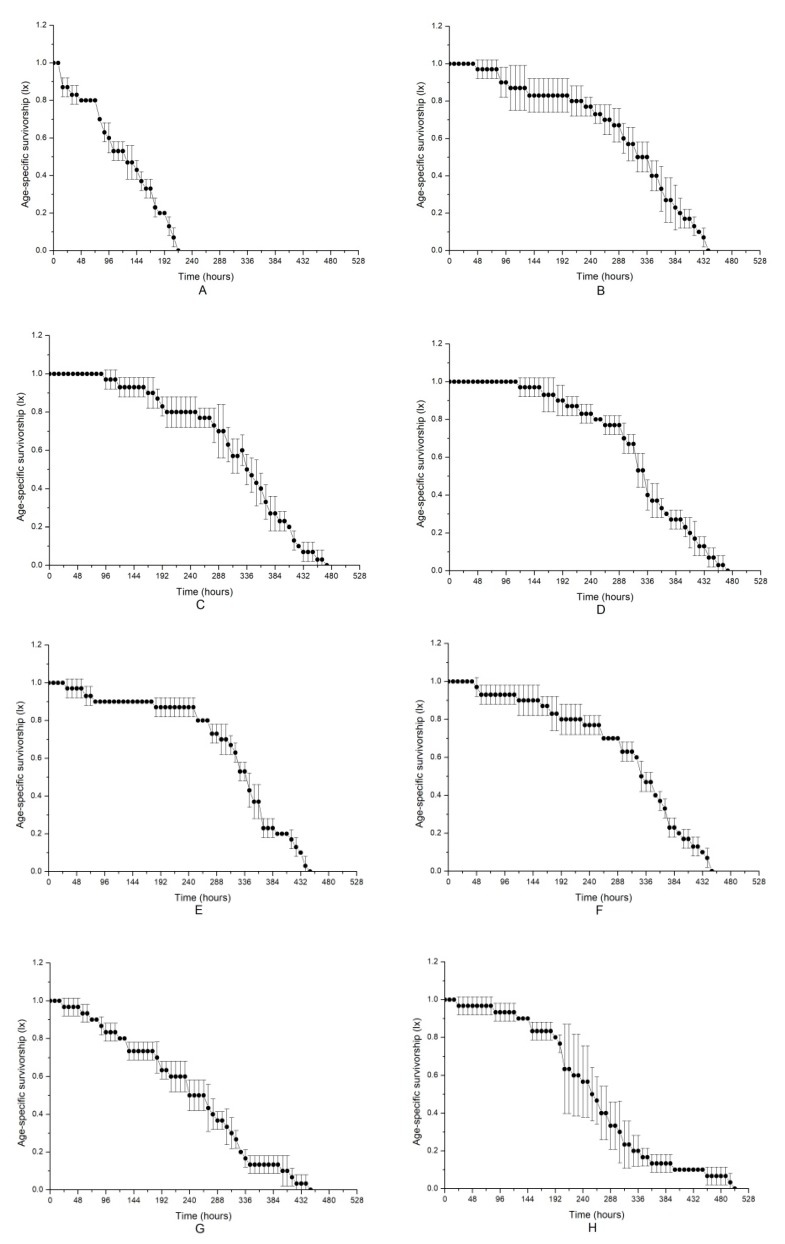

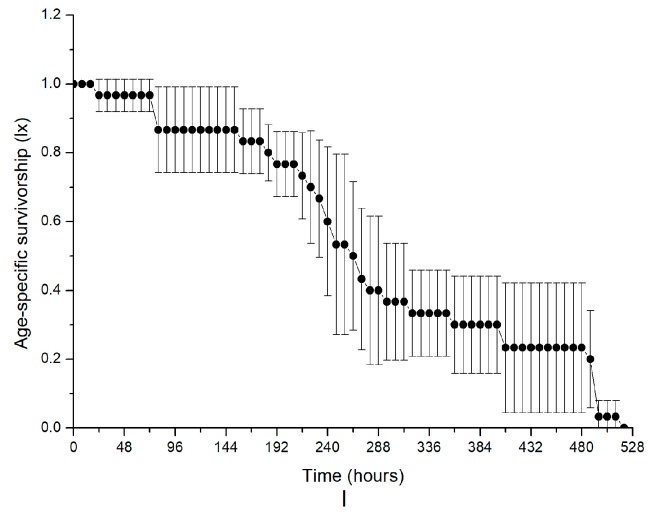
Allelopathic effects of different treatment fluids of *Karenia mikimotoi* on age-specific survivorship curves of rotifer *B. plicatilis.* The data are expressed as the mean ± SE (*n* = 3). (**A**) Group A; (**B**) Group B; (**C**) Group C; (**D**) Group D; (**E**) Group E; (**F**) Group F; (**G**) Group A (NAC); (**H**) Group B (NAC); (**I**) Group D (NAC).

The age-specific survivorships of *B. plicatilis* when exposed to different microalgal treatments are shown in Figure 2. The change in the age-specific survivorship in the control, which represented a normal change in survival, decreased steadily with increasing time and decreased to zero at approximately 450 h after treatment (Figure 2F). Similar to the alteration of population density, the most obvious damage to survival was observed in Group A, with the microalgal medium at a stationary phase with intact microalgal cells in it, and the survival period was shortened at a significance of *p* < 0.05 compared to that of the control. A decrease in the survival time in other treated Groups B–E was also found, but the difference was not as large as that in Group A; notably, the fluids in the exponential phase (Group B) were more altered than those in the stationary phase (Group D). NAC addition relieved the negatively allelopathic effects on the survival of *B. plicatilis*. Taking Group A as an example, NAC addition obviously increased age-specific survivorship compared to that without NAC addition (*p* < 0.05), although it was still much lower than that of the control. NAC treatment also alleviated the allelopathy of Group B and D. The results provided further evidence that the intact microalgal cells were necessary for the deleterious allelopathy induced by *K. mikimotoi* on the rotifer *B. plicatilis*, and ROS was not a key factor in the toxicity mechanism.

The alteration of the life table and the corresponding parameters were evaluated according to the changes in growth in differently treated groups (Table 2). Generally, the changes in all the parameters were altered in the following order: A > B > C > D > E, inferring that intact cell suspension exerted the most noxiously allelopathic effect on the co-cultured rotifer. Moreover, microalga in the exponential phase was more toxic compared to microalga in the stationary phase. Returning to the detailed information, all parameters in group A were much lower than those in the other treated groups and in the control (paired *t*-test, *p* < 0.05), among which the intrinsic rate of the population increased (*r_m_*), and the net reproductive rate (R_0_) was more sensitive to the treatment (Table 2). When NAC was added to the treated groups, all parameters were obviously higher than those without NAC addition, inferring the alleviation of the damage induced by the allelopathic effect of *K. mikimotoi*.

The parthenogenesis indicators in Table 3 showed the allelopathic effect of different treatment fluids of *K. mikimotoi* on the reproduction of *B. plicatilis*. The first spawning times of Groups A, B and D were significantly lengthened compared with that of the control (*p* < 0.05). However, the most obvious effects were observed in Group A, in which the first spawning time increased by approximately 32 h, while, in Groups B and C, the first spawning time was increased by 8 and 18 h, respectively. Other indicators of Group A were significantly smaller than that of the control (*p* < 0.05). In contrast, other parthenogenesis indicators of Groups (B–E) had no significant effects compared to Group F (*p* < 0.05). These results indicated that intact cell suspension exerted the most noxiously allelopathic effect on the rotifer. Moreover, the first spawning time of Group B was extended by 10 h compared with Group D, and other indicators of Group B were slightly lower than that of Group D, but the difference did not reach significance, inferring that microalga in the exponential phase were more toxic compared to microalga in the stationary phase. The addition of NAC significantly affected the allelopathic effect of algae on the rotifers. NAC addition shortened the first spawning time in Groups B and C (*p* < 0.05), and the first spawning time of group A was shortened from 84 h to 60 h, while the last spawning time was increased, and significance was found when compared to that of the control (Table 3). The results indicated that the reproductive injury induced in the co-cultured system was alleviated by NAC addition to some extent, but ROS seemed not to play a decisive role in the toxic effect of algae on rotifers.

### 2.2. Hemolytic Activity Analysis on Mussel and Rabbit Blood Cells

**Figure 3:**
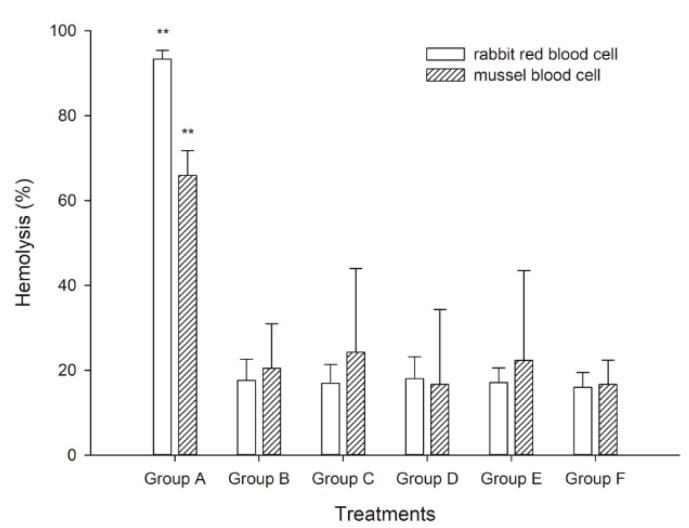
The hemolytic activity of different treatment fluids on rabbit and mussel blood cells. **: means extremely significant difference (*p* < 0.01, *n* = 3).

Two kinds of blood cells, rabbit red blood cells and mussel blood cells were applied to elucidate the hemolytic activity of *K. mikimotoi*. We found the highest hemolysis rate in Group A, which was approximately 98% and was approximately 3–4 times higher than that in the other treated groups (*p* < 0.01). In fact, the hemolytic rate in Groups B–E presented little difference in the present study. Interestingly, the hemolysis in rabbit cells was generally higher than that in mussel blood cells, which we speculated was because the components of the cells led to the observation. The rabbit blood cell was a single population, but the mussel blood cell contained different populations of hyalinocytes and granulocytes that played roles in immune function (Figure 3). This result consisted of the observation on population dynamics and reproductive changes with different microalgal fluid treatments, inferring that cell contact was not only noxious but also necessary for the allelopathic effect induced by *K. mikimotoi*. Mussel blood cells were also useful and effective in indicating the hemolytic activity of marine microalga.

## 3. Discussion

### 3.1. Is ROS Involved in the Allelopathy Induced by K. mikimotoi on the Rotifer B. plicatilis?

*B. plicatilis* were applied as a tool to assess the different levels of allelopathy induced by different treatments on *K. mikimotoi* in the present study. We observed significant toxic effects according to changes in the population dynamics, growth rate and survival curves, but the toxic damages were different in the groups with different treatments; the whole algal cell suspension significantly reduced the population density, the population growth rate was decreased by 0.87 compared with that of the control group, and the survival period was decreased with a significance of *p* < 0.05 compared to that of the control. The lysed cell suspension only inhibited the growth of population density, which reduced the growth rate by 0.12. In contrast, suspension without cells showed little toxic effects on the rotifers. Therefore, we found that microalgal culture medium intake cells presented the most significant inhibition on either the growth or reproduction of *B. plicatilis*, followed by a lysed culture medium treated with ultrasonication. The culture filtrate provoked a very limited impact on the target. The allelopathic test clearly demonstrated the existence of allelopathy induced by *K. mikimotoi*, and the microalgal cells seemed to be responsible for the present observation. These results were consistent with those of previous studies. For instance, *Heterocapsa circularisquama* was reported to exert noxious impacts on the co-culture organisms, and it was reported to secrete a kind of protein-like toxic substance onto the cell surface, which was an explanation for the toxicity [39]. Similar phenomena were found in other species of dinoflagellates, although the substances or the releasing pathways were species-specific [25,26,27]. Moreover, the released substances were usually unstable and easily disappeared or were depredated after releasing; therefore, the continuous release of the active substances was an effective way to sustain the concentration of allelopathic substances. The lysed cells acted differently; they lost the ability to secrete the substances, and the main source of allelopathy was from the surface of the sonicated cells, which was why the intact cell suspension exhibited the most obvious toxicity. Furthermore, we found that the culture filtrate or sonicated culture medium at different exponential or stationary growth phases demonstrated different levels of impact on the co-culture rotifer, and those in the exponential phase seemed more toxic compared to those in the stationary phase. We can see this from the first spawning time of the rotifers; in our study, we found that the first spawning time was significantly different between the exponential phase and the plateau phase of the lysed cell suspension, and the first spawning time of the exponential phase occurred much later than that of the plateau phase. Although there are no significant differences between the exponential phase of the lysed cell suspension and other indicators in the plateau phase, other indicators of the exponential phase had a downward trend compared with those of the plateau phase. This finding indicates that the culture medium in the exponential phase was more toxic than the culture medium in the plateau phase. It is speculated that the toxin content and toxin composition vary with environmental conditions and growth stages in culture, and the toxin content rapidly peaked during the early exponential phase and just as rapidly declined prior to the onset of the plateau phase. Similar phenomena have been found in previous studies. Cho et al. and Kim et al. have respectively reported that the total amount of toxins in a cell (toxin content) in *Alexandrium* spp. was highest during the mid-exponential phase of growth and decreased as the cultures aged [40,41].

*Chattonella polykrikoides* isolated in Korea was reported to produce O_2_ and H_2_O_2_, and the ROS-mediated ichthyotoxic mechanism of this dinoflagellate was proposed [42]. Other raphidophycean flagellates, such as *Heterosigma akashiwo*, *Olisthodiscus luteus* and *Fagus japonica*, were also found to produce ROS, and the generation of ROS seemed to be a common feature of raphidophycean flagellates [27,42,43]. Several lines of evidence suggested that some HAB species, including *K. mikimotoi*, produced ROS, such as superoxide anion (O_2_^−^), hydrogen peroxide (H_2_O_2_), and hydroxyl radical (OH^−^) and that these ROS might be involved in HAB-linked fish mortalities [44,45,46,47]. In the present study, to ascertain whether such an ROS-mediated toxic mechanism was responsible for the toxic mechanism of *K. mikimotoi*, bioassay experiments using *B. plicatilis* in the presence and absence of radical scavengers were conducted to detect ROS in *K. mikimotoi*. We thus added NAC, the ROS scavenger, to the culture system to eliminate the possible effects of ROS. We found that the normal microalgal culture medium without treatment still presented lethal effects on the rotifers, while that with sonic rapture treatment showed almost no inhibitory effect on rotifers with the NAC addition (Table 1, Table 2 and Table 3). It seemed that ROS produced by *K. mikimotoi* had no lethal toxicity on the rotifers and only inhibited the population growth of rotifers. Similar results were also derived from the population growth rate, age-specific survivorship curves, demographic parameters and parthenogenesis indicators after NAC was added, and the inhibition effect on rotifers of the normal microalgal culture medium was significantly (*p* < 0.05) decreased. However, the addition was still significantly (*p* < 0.05) lethal to rotifers. The inhibition effect on rotifers of the ultrasonic-ruptured cell suspension disappeared. As further illustrated, ROS was not involved in the toxicity mechanism induced by *K. mikimotoi* on *B. plicatilis*. Another interesting finding of the effect of *K. mikimotoi* on *B. plicatilis* was that the culture medium of *K. mikimotoi* with sonic rapture only led to the first spawning time of the rotifer that was delayed in the parthenogenesis endpoints test. Moreover, the delay was recovered with the addition of NAC. These results indicated that ROS contained in *K. mikimotoi* may only affect the reproductive capacity of *B. plicatilis*. However, further studies were required to identify the toxic mechanism of *K. mikimotoi*.

The hemolytic test was a simple and small-scale semiquantitative assay; it was useful not only for searching for toxic agents of algal cells but also for estimating its own potential toxicity. In the present study, we performed a hemolytic test on blood cells with differently treated algae fluids to explore the toxicity mechanism of *K. mikimotoi* and the differences in toxicity of different algae fluids. We found that culture mediums that contacted intact algal cells showed a more intense hemolytic activity (Figure 3). Furthermore, the cell-free supernatant and the ruptured cell suspension, which were incapable of killing rotifers, showed little hemolytic activity (Figure 3). These results suggested that the live cell-mediated hemolytic activity might be linked with the toxic effects on rotifers. Consistent with these findings, a previous study found that the toxic dinoflagellate *H. circularisquama* cell suspension causes marked hemolysis in rabbit erythrocytes in a cell density-dependent manner [48,49]. It seemed likely that the hemolytic substance on the cell surface of *H. circularisquama* was a toxin responsible for the cell-killing mechanism. Our results may support such a zooplankton-killing mechanism of dinoflagellate toxicity, which may also be applicable to the rotifer-killing mechanism of *K. mikimotoi*. *K. mikimotoi* caused membrane damage, leading to impaired membrane permeability in blood cells via direct algal cell contact and upon repeated exposure to a certain hemolytic substance located on the algal cell surface. The toxicity of *K. mikimotoi* to *B. plicatilis* was positively correlated with its hemolytic activity [50]. Based on these findings, it appeared that a hemolytic agent on the cell surface of *K. mikimotoi* may attack erythrocyte membranes through direct cell-to-cell contact, which in turn damages membrane structures. Thus, the possible toxicity mechanism of *K. mikimotoi* on the organisms in the marine ecosystem was hemolytic allelopathic. We also found an interesting point in this study in that the hemolysis on rabbit cells was generally higher than that on mussel blood cells. We speculated that this result was because the components of the cells led to the observation. The rabbit blood cell was a single population, but the mussel blood cell contained different populations of hyalinocytes and granulocytes that played roles in immune function (Figure 3). Although the hemolytic activity of *K. mikimotoi* on mussel blood cells was lower than that of rabbit blood cells, the hemolysis on mussel blood cells also showed strong hemolytic activity. Therefore, the mussel blood cell was also useful and effective in indicating the hemolytic activity of marine microalga. When combined and when considering the results in the present study, we presumed that *K. mikimotoi* was speculated to secrete allelopathic substances onto the cell surface, and direct cell contact was necessary for the allelopathic toxicity on *B. plicatilis*. ROS-independent hemolytic toxicity was assumed to be the explanation for the observed results, and the hemocytes of *M. edulis* could be used as good examples to elucidate the hemolytic activity induced by HABs.

### 3.2. The Possibility of Applying the Reproductive Changes in B. plicatilis as Biomarkers to Evaluate the Toxicity of HABs

Several endpoints had been proposed for assessing the effects of environmental toxicants on organisms but the most commonly used are lethality [51,52] and reproduction [53]. A comparison of the sensitivity between the two endpoints, survival and reproduction, was performed by Dhawan et al. [54]. Reproduction responses were found to be much more sensitive indicators of toxicity than lethality. These authors also noted that hormesis occurred more often in reproduction endpoints than survival. For example, Rao and Sarma showed that survivorship and the generation time (T) of the same rotifer species were not affected by the toxicant, while the net reproductive rate (R_0_) and intrinsic rate of increase (*r_m_*) were affected negatively [55]. Based on this conclusion, the indicators of survival and reproduction (Table 3 and Table 4) were compared to evaluate the toxic difference in algae suspension under different treatment conditions in our study. We found that whole cell algae fluid caused a significant change in all parameters of the life table and in the parthenogenetic indicators, which meant that the toxicity of the whole cell suspension not only caused significant changes in the reproductive indicators but also caused significant changes in the survival indicators. In contrast, the lysed cell suspension only caused significant changes in the intrinsic growth rate (*r_m_*) and the first spawning time of reproductive parameters, which meant that the lysed cell suspension permitted survival but impaired the reproduction of *B. plicatilis*. The culture filtrate had a small impact on survival and reproductive parameters. The data here demonstrated that most of the life table parameters of the rotifer *B. plicatilis* were more sensitive to the whole cell suspension than to lysed cell suspension and the culture filtrate, and the whole cell suspension was more toxic to *B. plicatilis* than the lysed cell suspension and culture filtrate. These results further demonstrated that the whole cell suspension had the most allelopathic effect on the rotifers, and direct contact with whole cells was a necessary condition for the allelopathic toxicity on *B. plicatilis*, followed by the lysed cells suspension. The culture filtrate provoked the smallest impact on the rotifers. It is speculated that the whole cell surface continued to secrete allelopathic substances until this toxic substance reached the death concentration of the rotifers. However, the lysed cells lost the ability to secrete allelopathic substances; thus, the allelopathic substances of the original cells only caused sublethal toxicity effects but did not cause the death of the rotifers [56]. Furthermore, direct contact with whole cells was a necessary condition for the allelopathic toxicity on *B. plicatilis*. This conclusion was also consistent with our previous speculation.

## 4. Conclusions

We found substantial hemolytic toxicity in the treatment of the intact cell suspension, which presented the most significant growth inhibition, including lethality, on *B. plicatilis*, and the addition of NAC could not change the growth or reproduction patterns. These findings provided evidence that intact *K. mikimotoi* secrete allelopathic substances onto the cell surface, that direct cell contact was necessary for the allelopathic toxicity on *B. plicatilis* and that ROS-independent allelopathy was assumed to be the explanation for the toxic mechanism of *K. mikimotoi* on the rotifers. What should be emphasized was that the hemocytes of *M. edulis* could be used as a good example to elucidate the hemolytic activity induced by HABs.

## 5. Materials and Methods

### Organism Cultivation

*K. mikimotoi* was kindly provided by the Algal Center of the Ocean University of China. The algae were grown in closed Erlenmeyer flasks with modified f/2 media [57] at 22 ± 1 °C 80 μmol photon m^−2^ s^−1^ with a 12 h light: dark cycle in illuminating incubators. The initial pH and salinity of the culture medium were adjusted to 8.0 ± 0.02 and 30, respectively [58]. Flasks containing the microalgae were shaken manually twice at set times for one day. The microalgae were cultivated to an exponential growth phase for use. 1 mL sample was collected daily and was preserved in Lugol’s solution to estimate microalgal growth by directly counting cell numbers and using a hemocytometer under an optical microscope (Motic SFC-18, Motic China Co. Ltd., Xiamen, China).

The rotifer *B. plicatilis* used in this experiment was obtained by hatching resting eggs that were provided by the College of Fisheries of the Ocean University of China. The hatching conditions were the same as those in our previous description [37,59]. After hatching, the rotifers were cultivated in an illuminating incubator with a 12 h: 12 h light: dark cycle at (25 ± 1) °C, 80 μmol photons/(m^2^·s). The rotifers were pre-cultured for at least two weeks before the formal experiment. Sterilized seawater was used for the cultivation of the rotifers, and the salinity and pH were adjusted to 30 and 8.1, respectively. The rotifers were daily fed with *Chlorella* sp. at a concentration of 1.0 × 10^6^ cells/mL, and the cultivation conditions for the bait microalga were the same as those of *K. mikimotoi*. The rotifers were acclimated to the experimental conditions for approximately 48 h by inoculating them in tissue culture plates containing 24 wells at a density of 10 ind./mL. The active and strong female individuals with eggs were chosen and then were placed in other tissue culture plates under the same conditions to observe the number of eggs hatched. Neonates that hatched within 2 h were used for the follow-up experiments [38].

## 6. Experimental Design

### 6.1. Allelopathic Effects of K. mikimotoi on the Growth of B. plicatilis

*K. mikimotoi* was cultivated to the exponential and static phases, and the culture filtrate and culture medium were prepared according to the method described by [57], *K. mikimotoi* grown in f/2 media enrichment at a static growth phase was prepared and referred to as Group A in this study. Another microalgal medium that was sonicated with an ice bath (600 W, intermittent broken, broken 5 s, intermittent 5 s) was combined with a lysed cell suspension in the experiment and was referred to as either Group B or Group D when it was in the exponential or static growth phase, respectively. The f/2 medium was also added into each group to eliminate possible nutrient limitation. The microalga-free culture medium was prepared by filtering the microalgal medium during the exponential or static phase through the GF/F membrane (Φ = 0.22 μm) and then was enriched with the f/2 medium [57]; these two treated groups were designated as Group C and E, respectively. The cell density of *K. mikimotoi* in the algae fluid required for each experimental group was 10^6^ cells mL^−1^. The group grown in sterilized seawater with f/2 enrichment was used as the control (Group F).

A four-day population growth test of the rotifers was applied to estimate the toxic effect of *K. mikimotoi* with different treatments. Neonates (<2 h) were placed in 24-well tissue culture co-cultured with the microalgal treatment of A–F. The number of neonates born and the number of surviving adults were counted under a microscope every 24 h, and the dead ones were removed from the culture system. The population growth rate (*r*, day^−1^) for each group was calculated according to the following Equation (1) [60]:

(1) $$\;r=\left(\ln N_{t}-\ln N_{0}\right)/t\;$$

where *N_t_* (individuals/mL) denoted the density of individuals at time *t* (day), *N*_0_ is 10 individuals/mL, and *t* is 4 days.

Ten neonates (<2 h) were placed in 24-well tissue culture plates co-cultured with different microalgal treatment fluids of *K. mikimotoi*. Following inoculation, we counted the number of neonates born, the surviving adults, and the eggs produced every 4 h during the first 72 h and every 8 h during the rest of the experiment. Then, the dead individuals and neonates were removed when they were found. The survival rate and the life table parameters including the net reproductive rate (R_0_), intrinsic rate of growth (*r_m_*), generation time (T), life expectancy (E_0_) and intrinsic growth rate (λ) were recorded according to previous documents [60,61,62]. This method was described by Hitchcock et al. Krebs et al. and Pianka et al. Other cultivating conditions were the same as those noted above, without other descriptions [61,62,63].

Regarding the analysis of reproductive toxicity, 10 neonates (<2 h) were placed in each plate of 24-well tissue culture plates and exposed to different microalgal fluids treatments. The experimental system and analyzing methods were the same as those described above. The first spawning time, the last spawning time, the average number of eggs, the average number of larva and the hatchability were recorded.

NAC is a type of ROS inhibitor. NAC addition was performed in the experimental systems set up in 2.1 to elucidate whether the allelopathic effect was ROS dependent or independent. NAC, at a concentration of 1 mM, was added to the treated groups A–E. Parameters related to the life table were recorded simultaneously according to the method described above.

### 6.2. Analysis of the Hemolytic Activity of K. mikimotoi

The rabbit blood cells were obtained from Beijing Solarbio Science & Technology Company. The rabbit blood cells were stored in the dark at 4 °C to avoid photolysis. We innovatively tried to use the hemocytes of the blue mussel *Mytilus edulis* to assess the hemolytic activity of *K. mikimotoi* in the present study. The hemocytes of *M. edulis* were collected from the posterior adductor according to our previous study, as described by Jiang et al. [64], and the extracted hemocytes were kept on ice for further analysis. Different treatment fluids of *K. mikimotoi* were mixed with the suspension of rabbit red blood cells (4%, Solarbio) with a ratio of 1:1 and mussel blood cells with a ratio of 1:4 in the centrifuge tube (1.5 mL). The number of blood cells was counted in the blood cell counting chamber. After incubation for 5 h at 26 °C under illumination from a fluorescent lamp (200 μmol photons/(m^2^·s)), the number of blood cells was counted again. Then, we obtained the hemolysis rate by the following Equation (2) [65]:

(2) $$\;\mathrm{Hemolysis}\;\mathrm{rate}=\left(C_{0}-C_{t}\right)/C_{0}\times 100\%\;$$

where *C_t_* (cell/mL) denotes the density of blood cells at time *t* (hour), *C*_0_ is the density of blood cells at the beginning, and *t* is 5 h.

### 6.3. Statistical Analysis

SPSS Statistics 17.0 and Excel were used to analyze the data. The difference between the test and control results was analyzed statistically with one-way analysis of variance (one-way ANOVA) and multiple comparisons (LSD test) with the significance set at *p* < 0.05. The mean values and standard errors were calculated from the different replicates of each treatment (*n* = 3).

## Figures and Tables

**Table 1:** Changes in the population growth rate (*r*) of rotifers co-cultured with different microalgal treatments with and without NAC addition.

Treatment Fluids	The Population Growth Rate *r*
group A	−0.3685 ± 0.1493 ^d^
group B	0.3903 ± 0.0066 ^b^
group C	0.5045 ± 0.0122 ^a^
group D	0.3895 ± 0.0021 ^b^
group E	0.5046 ± 0.0104 ^a^
group F	0.5080 ± 0.0078 ^a^
group A (+NAC)	−0.1452 ± 0.0397 ^c^
group B (+NAC)	0.5175 ± 0.0125 ^a^
group D (+NAC)	0.5091 ± 0.0081 ^a^

Note: Values were means ± SE (Standard Error) of three replicates. Values followed by the same letter are not significantly different at a probability level of 0.05, as determined by Duncan’s multiple range test.

**Table 2:** Demographic parameters of *B. plicatilis* exposed to different treatment fluids of *K. mikimotoi*.

Parameter	R_0_	T	*r_m_*	E_0_	λ
Group A	2.0667 ± 0.3399 ^b^	5.1130 ± 0.1568 ^d^	0.1386 ± 0.0322 ^e^	4.5000 ± 0.2449 ^c^	1.1492 ± 0.0367 ^e^
Group B	12.6667 ± 2.2005 ^a^	7.0630 ± 0.2211 ^ab^	0.3571 ± 0.0167 ^c^	12.0000 ± 1.2570 ^ab^	1.4294 ± 0.0237 ^c^
Group C	13.5667 ± 1.6977 ^a^	7.2136 ± 0.2958 ^a^	0.3602 ± 0.0036 ^c^	12.6333 ± 0.7409 ^a^	1.4336 ± 0.0052 ^c^
Group D	15.1000 ± 2.6470 ^a^	6.8249 ± 0.3748 ^ab^	0.3953 ± 0.0144 ^bc^	13.0333 ± 0.2357 ^a^	1.4850 ± 0.0214 ^c^
Group E	13.6333 ± 2.9318 ^a^	6.5161 ± 0.6245 ^b^	0.4010 ± 0.0456 ^bc^	12.7333 ± 0.0471 ^a^	1.4948 ± 0.0672 ^bc^
Group F	15.6333 ± 2.4226 ^a^	6.4576 ± 0.2978 ^bc^	0.4239 ± 0.0088 ^b^	12.2333 ± 0.4497 ^ab^	1.5280 ± 0.0135 ^bc^
Group A(NAC)	3.3667 ± 0.3771 ^b^	5.2338 ± 0.3310 ^cd^	0.2315 ± 0.0231 ^d^	9.3000 ± 0.2944 ^b^	1.2609 ± 0.0290 ^d^
Group B(NAC)	13.7000 ± 0.5354 ^a^	5.3092 ± 0.0751 ^cd^	0.4929 ± 0.0106 ^a^	10.4000 ± 0.9899 ^b^	1.6372 ± 0.0174 ^a^
Group D(NAC)	12.9667 ± 0.9463 ^a^	5.8012 ± 0.1915 ^c^	0.4413 ± 0.0038 ^b^	11.5667 ± 2.4074 ^ab^	1.5548 ± 0.0059 ^b^

Note: Values are means ± SE (Standard Error) of three replicates. Values followed by the same letter are not significantly different at a probability level of 0.05, as determined by Duncan’s multiple range test.

**Table 3:** Parthenogenesis indicators in rotifer exposed to different treatment fluids of *K. mikimotoi*.

Groups	The First Spawning Time	The Last Spawning Time	The Average Number of Eggs	The Average Number of Larvae	Hatchability
Group A	84.000 ± 0.001 ^a^	174.667 ± 11.624 ^c^	20.667 ± 2.404 ^b^	26.667 ± 3.712 ^b^	79.085 ± 10.519 ^b^
Group B	70.667 ± 1.333 ^b^	308.000 ± 16.000 ^a^	126.667 ± 15.560 ^a^	133.000 ± 15.822 ^a^	95.153 ± 1.850 ^a^
Group C	60.000 ± 4.619 ^c^	300.000 ± 16.653 ^ab^	135.667 ± 12.005 ^a^	142.667 ± 14.518 ^a^	95.384 ± 1.434 ^a^
Group D	56.000 ± 4.000 ^cd^	310.667 ± 17.487 ^a^	151.000 ± 18.717 ^a^	153.000 ± 19.088 ^a^	98.724 ± 0.308 ^a^
Group E	53.333 ± 1.333 ^cd^	284.000 ± 36.661 ^ab^	136.333 ± 20.731 ^a^	147.667 ± 17.629 ^a^	91.834 ± 4.234 ^a^
Group F	52.000 ± 2.309 ^d^	305.333 ± 26.667 ^a^	156.333 ± 17.130 ^a^	161.333 ± 15.836 ^a^	96.725 ± 2.170 ^a^
Group A (NAC)	60.000 ± 4.000 ^c^	316.000 ± 20.000 ^a^	33.667 ± 2.667 ^b^	38.000 ± 1.155 ^b^	88.397 ± 4.736 ^ab^
Group B (NAC)	54.667 ± 1.333 ^cd^	226.667 ± 7.055 ^bc^	148.667 ± 5.044 ^a^	156.333 ± 6.119 ^a^	95.187 ± 2.268 ^a^
Group D (NAC)	52.000 ± 2.309 ^d^	240.000 ± 16.000 ^b^	129.667 ± 6.692 ^a^	139.000 ± 2.082 ^a^	93.187 ± 3.407 ^a^

Note: Values are the means ± SE (Standard Error) of three replicates. Values followed by the same letter are not significantly different (at a probability level of 0.05), as determined by Duncan’s multiple range test.

**Table 4:** The experimental conditions of different groups.

Group	Definition	Treatment Conditions	Growth Phase
Treatment group	Group A	Whole cell suspension	Plateau phase
	Group B	Lysed cell suspension	Exponential phase
	Group C	Cell-free culture filtrates	Exponential phase
	Group D	Lysed cell suspension	Plateau phase
	Group E	Cell-free culture filtrates	Plateau phase
Control group	Group F	Sterilized seawater	
